# The validity of parental reports on motor skills performance level in preschool children: a comparison with a standardized motor test

**DOI:** 10.1007/s00431-017-3078-6

**Published:** 2018-02-09

**Authors:** Annina E. Zysset, Tanja H. Kakebeeke, Nadine Messerli-Bürgy, Andrea H. Meyer, Kerstin Stülb, Claudia S. Leeger-Aschmann, Einat A. Schmutz, Amar Arhab, Valentina Ferrazzini, Susi Kriemler, Simone Munsch, Jardena J. Puder, Oskar G. Jenni

**Affiliations:** 1 Child Development Center, University Children’s Hospital, Steinwiesstrasse 75, 8032 Zürich, CH Switzerland; 2 Children’s Research Center, University Children’s Hospital Zurich, Zurich, Switzerland; 3 Department of Clinical Psychology and Psychotherapy, University of Fribourg, Fribourg, Switzerland; 4 Service of Endocrinology, Diabetes and Metabolism, Lausanne University Hospital, Lausanne, Switzerland; 5 Department of Psychology, University of Basel, Basel, Switzerland; 6 Epidemiology, Biostatistics and Prevention Institute, University of Zurich, Zurich, Switzerland; 7 Service of Pediatric Endocrinology, Diabetology and Obesity, Lausanne University Hospital, Lausanne, Switzerland

**Keywords:** Motor skills, Parent report, Preschoolers, Questionnaire, Splashy

## Abstract

Motor skills are interrelated with essential domains of childhood such as cognitive and social development. Thus, the evaluation of motor skills and the identification of atypical or delayed motor development is crucial in pediatric practice (e.g., during well-child visits). Parental reports on motor skills may serve as possible indicators to decide whether further assessment of a child is necessary or not. We compared parental reports on fundamental motor skills performance level (e.g., hopping, throwing), based on questions frequently asked in pediatric practice, with a standardized motor test in 389 children (46.5% girls/53.5% boys, M age = 3.8 years, SD = 0.5, range 3.0–5.0 years) from the Swiss Preschoolers’ Health Study (SPLASHY). Motor skills were examined using the Zurich Neuromotor Assessment 3–5 (ZNA3–5), and parents filled in an online questionnaire on fundamental motor skills performance level. The results showed that the answers from the parental report correlated only weakly with the objectively assessed motor skills (*r* = .225, *p* < .001).

*Conclusion*: Although a parental screening instrument for motor skills would be desirable, the parent’s report used in this study was not a valid indicator for children’s fundamental motor skills. Thus, we may recommend to objectively examine motor skills in clinical practice and not to exclusively rely on parental report.

**Table:** 

**What is Known:** *• Early assessment of motor skills in preschool children is important because motor skills are essential for the engagement in social activities and the development of cognitive abilities. Atypical or delayed motor development can be an indicator for different developmental needs or disorders.* *• Pediatricians frequently ask parents about the motor competences of their child during well-child visits.*
**What is New:** *• The parental report on fundamental motor skills performance level used in this study was not a reliable indicator for describing motor development in the preschool age.* *• Standardized examinations of motor skills are required to validly assess motor development in preschoolers.*

## Introduction

Motor skills are interrelated with a number of developmental domains such as cognition, perception, language, and social and physical development [1, 2, 6, 7, 9, 22]. For example, Cameron et al. [5] reported that in 3–4-year-old motor skills correlated positively with performance in a Kindergarten achievement test, including language skills (e.g., reading, vocabulary, and phonological awareness), and mathematical problems. Michel et al. [19] found that 5–7-year-old children with impaired motor skills showed lower pre-academic skills and lower performance in inhibition tasks compared to children without motor impairments.

Furthermore, several studies showed that fundamental motor skills (FMS) are essential for the engagement in physical activities and to discover the environment [2, 24]. FMS include locomotor (e.g., moving from place to place: walking, running, jumping, skipping, hopping, sliding, etc.) and object control skills (e.g., throwing, catching, kicking) [8, 24]. Therefore, the competence in FMS is linked to health-related outcomes such as cardiorespiratory fitness, muscular strength, and body weight [16, 21]. Stodden and co-workers stated that children who perceive their motor competence as low engage less in physical activity and, thus, bear a higher risk of becoming unfit and obese [24]. Both reduced physical activity and high body weight further promote low perception of motor competence which will eventually result in even lower motor competence [24]. As a result, children find themselves in a “negative spiral of disengagement” [24]. In fact, less engagement in physical activities can also affect the social interaction with peers negatively, especially in the preschool age, and may lead to social exclusion [1, 23]. Smyth and Anderson [23] found that children with developmental coordination disorder (DCD) spent more time alone or were more watching other children play compared to children without motor difficulties. These authors discussed that children with DCD might be excluded first from physical and then from social games. Moreover, potential co-occurring difficulties (e.g., cognitive deficits, language impairment, etc.) might have an additional influence on the exclusion. However, actual causality remains open.

To avoid this negative spiral, it is important to assess motor performance early enough so that therapeutic intervention and support for the child may be introduced. Thus, the evaluation of FMS performance level in early childhood and the identification of atypical or delayed motor development is crucial in pediatric practice. In fact, pediatricians regularly assess FMS performance level during well-child visits by asking parents whether their child can already perform a certain task (e.g., climbing stairs, riding a bicycle, swimming) [3, 11]. Parental reports are an attractive option for receiving information about the development of the child. They are time and cost effective, and easy to implement. Parents have knowledge of the unaffected behavior and the skills of their children, whereas in clinical practice motivation and cooperation of the child may lead to ambiguous evaluation. Although evidence exists that parents provide valid and reliable reports regarding early motor milestones during the first years of life [4, 15, 17], we do not know whether FMS performance level reported by parents during the preschool years ultimately reflect the child’s performance in a standardized motor test. To our knowledge, there is no study examining parental reports on motor skills in typically developing preschool children (which was also stated in [20]). In pediatric practice, it would be beneficial to know whether questions on daily motor activities of the child correlate with motor skills measured by a standardized test. Questions about daily motor activities aim to identify indicators for motor skills performance level. So far, it has not been examined whether these questions deliver some additional information on motor development.

Thus, we constructed a 6-item questionnaire of FMS based on questions frequently asked in pediatric practice [3, 11] and compared the answers with objectively measured FMS performance level using the Zurich Neuromotor Assessment 3–5 (ZNA3–5), a standardized test instrument with good psychometric properties. Our aim was to evaluate whether a parental report on FMS performance level observed in everyday activities can deliver valid data about the level of motor skills development in the preschool age as measured by a standardized test procedure.

## Materials and method

### Participants

Our analysis included 389 children between 3 and 5 years of age (181 girls/208 boys, M age = 3.8 years, SD = 0.5, range 3.0–5.0 years). The data presented here were collected within the Swiss Preschoolers’ Health Study (SPLASHY) that investigated typically developing preschool children in 84 child care centers [18]. Originally, 476 children participated in the SPLASHY study. For this analysis, we excluded children below the age of 3 years and above the age of 5 years. From this sample (*n* = 417), 24 parents did not fill out the motor questionnaire. Out of the remaining 393 parents, 389 parents answered at least three items, so that a total parental report score could be calculated.

### Measurements

Motor skills were examined using the ZNA3–5 [12]. The ZNA3–5 is based on the original ZNA for children older than 5 years (ZNA5–18; [13, 14]) and is a well-standardized motor test instrument. The ZNA3–5 has a moderate to high intra-observer (kw = 0.56–1.00) and inter-observer (kw = 0.42–0.99) reliability, while test-retest reliability is lower in some tasks (0.35–0.84) [12].

Fundamental motor skills were measured with static balance (standing on one leg) and dynamic balance (walking on a straight line, hopping on one leg, side-to-side jumping, and running). The instruction for static balance was “stand on your right/left leg as long as you can”. Timing started when the child lifted one foot off the floor and stopped when the child touched the floor with the lifted foot, or shifted the foot of the standing leg more than 2 cm, or when the time limit of 30 s was reached. Instructions for the dynamic balance tasks were the following: (1) Walking on a straight line: the child was asked to walk on the cord by putting one foot in front of the other. The heel of the anterior foot had to touch the toes of the foot behind. A qualitative score was given from 0 to 4 (0 = Perfect performance, heel touching toes; 1 = Distance between the two feet, feet straight; 2 = Feet not straight and/or misses the line 1–3 times; 3 = Feet perpendicular and/or does not touch the line > 3 times; 4 = Not able to walk with both feet on the line), (2) Hopping on one leg: the child has to hop as many times as possible on one leg, next to the cord. The task was done for each leg, and two trials for each leg were given. A qualitative score was given from 0 to 4 (0 = Can hop on both legs more than 7 times; 1 = Can hop on only one leg more than 3 times; 2 = Can hop on both legs from 1 to 3 times; 3 = Can hop on only one leg from 1 to 3 times; 4 = Cannot hop on either leg), (3) Side-to-side jumping: the child was asked to stand beside the cord and to jump forth and back over the cord sideways while keeping the feet together. A qualitative score was given from 0 to 4 (0 = Perfect performance, very smooth jumping; 1 = Jumping is correct but not very smooth; 2 = Touchdown with two feet at the same time, jumping very stiff; 3 = Total body involvement, poor coordination in relation to the line direction; 4 = Jumping about but not in relation to the line) and (4) Running: the child had to run 20 m around the chairs (5 × 4 m). A qualitative score was given form 0 to 4 (0 = Rolling motion of feet with adjustment of upper body; 1 = Rolling motion of feet, stiff upper body; 2 = Running with partial rolling motion of feet; 3 = Running without any rolling motion of feet; 4 = Cannot run (no flight phase)). For the analyses, all ZNA3–5 performance was expressed as standard deviation scores (SDS) calculated from age- and sex-adjusted normative values. Positive values are corresponding to above average performance and negative values to below average performance.

Parents filled in an online questionnaire (see abstract) containing questions about swimming, climbing stairs, hopping, riding, balancing, and throwing (Table 1). For each FMS item, the parents had to rate the stage of development. Responses were combined into three categories: 0–1–2 (Table 1). A sum score for the parental FMS questionnaire (parental FMSQ) was calculated by taking the average score across the six items (if at least three items were answered), multiplied by the amount of all items.

**Table 1:** Items and descriptive statistics of parental report on FMS assessed by questionnaire (frequency distribution)

Points	Questionnaire items	Frequency in %				*n*
		1st T	2nd T	3rd T	All	Total
	*Swimming*					375
0	Cannot swim	46.3	30.5	27.6	34.1	
1	Can swim with swimming aid	53.7	66.2	63.8	61.9	
2	Can swim without swimming aid	0.0	3.3	8.6	4.0	
	*Climbing stairs*					366
0	Cannot climb stairs or only by crawling on all fours	1.0	0.7	0.0	0.5	
1	Can climb stairs in upright posture, but holds the banister	13.3	5.7	3.3	7.1	
2	Can climb the stairs in upright posture without holding the banister	85.7	93.6	96.7	92.3	
	*Jumping*					376
0	Cannot jump	2.9	3.4	0.0	2.1	
1	Can jump with both legs	58.1	43.0	16.4	38.6	
2	Can jump on one leg	39.0	53.7	83.6	59.3	
	*Riding*					387
0	Cannot ride bicycle/scooter/tricycle/tractor with support wheels	1.9	0.6	1.6	1.3	
1	Can ride tricycle/scooter/balance bicycle/bicycle with support wheels	93.5	74.8	50.0	72.1	
2	Can ride a bicycle without support wheels	4.6	24.5	48.4	26.6	
	*Balance*					261
0	Can neither balance forwards or backwards on a bar	19.3	11.4	1.0	9.2	
1	Can balance forwards on a bar (at least 8 steps)	70.2	77.1	71.7	73.6	
2	Can balance forwards and backwards on a bar (at least 8 steps)	10.5	11.4	27.3	17.2	
	*Throwing*					355
0	Cannot catch a ball	7.0	6.4	1.7	5.1	
1	Can catch or throw targeted	54.0	38.6	19.1	36.6	
2	Can catch and throw targeted	39.0	55.0	79.1	58.3	

Descriptive statistics are presented for the entire sample (all), and for the sample divided in three tertile groups. *T* tertile

### Statistical analyses

Statistical analyses were performed using SPSS (IBM, SPSS; Version 22.0, Chicago, IL, USA). Descriptive statistics were calculated by means ± standard deviations for continuous variables and percentages for categorical variables. The main outcome variables ZNA scores and sum parental FMSQ score were normally distributed. For parental FMSQ, sex effects were tested with the Mann-Whitney *U* Test and age effects with Spearman’s rank order correlations. Corresponding effect sizes were calculated. SDS scores for ZNA were sex and age-adjusted and therefore these effects were no more examined. The relationship between ZNA outcome and parental FMSQ outcome was investigated using partial correlation, with age and sex as control variables. Furthermore, the sample was divided in three tertiles by age to test whether parental report delivers reliable information for all age groups in the preschool age: first tertile *n* = 129, *M* = 3.3 years, range 3.0–3.5; second tertile *n* = 130, *M* = 3.8 years, range 3.5–4.1, and third tertile *n* = 130, *M* = 4.4 years, range 4.1–5.0. Partial correlations were compared with Spearman’s rank order correlations, which are more adequate for ordinal variables but do not allow to include control variables. Correlations from both analyses were very similar in magnitude and significance level (Table 2). Therefore, only partial correlations controlled for age and sex are discussed.

**Table 2:** Association between parental fundamental motor skill questionnaire (FMSQ) and ZNA motor skills scores; above the diagonal, correlation coefficients controlled for age and sex are presented; under the diagonal, spearman correlation coefficients are presented

	1	2	3	4	5	6	7	8	9	10	11	12	13
ZNA3–5													
1. Static balance		.326***	.144*	.207***	.120*	.321***	.007	.121*	.072	.163**	.006	.064	.137*
2. Walk on a line	.307***		.225***	.212***	.172**	.660***	.070	.133*	.158**	.069	.010	.055	.151**
3. Jump side-to-side	.165**	.212***		.137*	.121*	.618***	.018	.065	.094	.075	− .095	.052	.097
4. Hop on one leg	.178**	.190***	.136*		.099	.593***	− .059	.111	**.**215***	.130*	.045	.004	.154*
5. Run	.080	.116*	.133*	.088		.584***	− .006	.036	.094	.072	.224**	.040	.145**
6. Total dynamic balance	.304***	.621***	.590***	.584***	.544***		.016	.123*	.228***	.138*	.087	.066	.225***
FMSQ													
7. Swim	.039	.063	.025	− .040	− .034	.027		.009	.079	.179**	.028	− .004	.504***
8. Stairs	.147*	.137*	.078	.144*	.016	.166**	.036		.198***	.061	.004	.030	.346***
9. Jump	.086	.165**	.095	.238**	.025	.240***	.166**	.195***		.145**	.190**	.162**	.603***
10. Ride	.172**	.066	.072	.141*	.008	.131*	.233**	.095	.265**		− .001	.022	.462***
11. Balance	− .011	.018	− .095	.048	.123	.080	.082	.040	.273***	.093		− .009	.453***
12. Throw	.074	.070	.044	.023	− .017	.072	.048	.052	.241***	.158**	.068		.510***
13. Sum FMSQ	.142*	.131*	.165**	.178**	.080	.304***	.515**	.304***	.678***	.573***	.494***	.567***	

Significant correlations *p < .05, **p < .01, ***p < .001

## Results

**Fig. 1:**
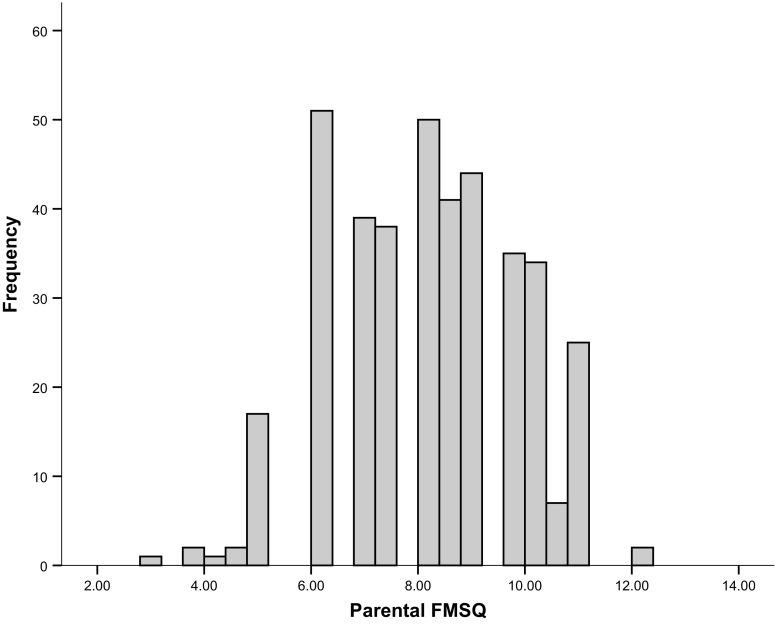
Frequency distribution of the parental report sum score

Parental FMSQ scores ranged from 3 to 12 with a mean sum score of Median = 8.00 (SD = 1.80) (Fig. 1). Frequencies of each answer category per items are shown in Table 1. There was no sex difference in the sum score of the parental FMSQ, (*p* = .31), while we found small sex differences for the items riding, *U* = 16,273.0, *p* < .05 (effect size *r* = .14), and throwing, *U* = 13,781.0, *p* < .05, (effect size *r* = .12), with boys showing a higher score on both items. Furthermore, there was a strong age effect, *r* = .506, *p* < .001; older children scored higher than younger children. The internal consistency between the six FMSQ items was expressed by a Cronbach alpha of .50.

The questionnaire was mainly filled out by mothers (84.3%, in 14.7% exclusively by the fathers). We compared the children included in the analyses with the 24 excluded children without motor questionnaire data; they did not differ in age, sex, SES, and the tested ZNA tasks (*p* > .05). ZNA scores were age and sex adjusted, therefore no corresponding effects can be reported.

Overall, the parental FMSQ correlated weakly to moderately with the ZNA total dynamic balance tasks, *r* = .225, *p* < .001 and weakly with static balance *r* = .137, *p* < .05. The FMSQ item jump revealed the strongest correlations with ZNA outcomes (Table 2); significant correlations were found between jumping and walking on a straight line, hopping on one leg, and total dynamic balance (*r* = .158–.228) (Table 2). The three items—stairs, ride, and balance correlated with several tasks from the ZNA, while the items swim and throw did not correlate with any tasks from the ZNA.

The same partial correlations between ZNA motor tasks and the FMSQ items were performed for three different age groups. Correlations for parental FMSQ sum and ZNA total dynamic balance were nearly the same in all age groups (*r* = .196–.284, *p* < .05). As for the overall analysis, the item jump from the FMSQ correlated most frequently and strongest with ZNA tasks in all age groups (*r* = .220–.325), while swim and throw were not correlated with any of the ZNA tasks. Between the three age group, differences occurred, but no systematic differences in amount or magnitude of significant correlation was observed. Correlations only significant in a single age group were the following: only in the youngest group static balance (ZNA) was correlated with FMSQ sum (*r* = .307, *p* = .003) and stairs (*r* = .276, *p* = .009), and, only in the middle group, the item stairs (FMSQ) and walking on a straight line (ZNA) were correlated (*r* = .232, *p* = .020). The item balance (FMSQ) and running (ZNA) were correlated in the first and second group (*r* = .290/.265, *p* < .05).

## Discussion

The findings of this analysis of the SPLASHY data showed that the rating of FMS performance level by parents correlated weakly to moderately with standardized measured FMS performance level in the preschool age. Out of the six questioned motor skills four items—climbing stairs, jumping, riding, and balancing—correlated weakly with measured motor skills. Swimming and throwing did not correlate with any motor tasks from the ZNA.

Climbing stairs, jumping, and riding from the FMSQ were correlated weakly with measured total dynamic balance and single tasks from the ZNA static balance, walking on a straight line, and hopping on one leg. The item jump from the FMSQ correlated slightly stronger with ZNA outcomes than climbing stairs and riding; still, the correlation found between jump and the corresponding ZNA tasks hopping on one leg was weak to moderate. No correlations were found between FMSQ items and side-to-side jumping. Balance from the FMSQ was correlated only with running. This is surprising because the performed ZNA tasks, walking on a straight line, side-to-side jumping, and hopping on one leg substantially include balancing skills, even though, more than running. Another unexpected result was that static balance, measured separately, did also not correlate with balance from the FMSQ. A reason might be that 33% of the parents did not know whether their child can balance, so for the item balance fewer children were included, which can result in a power problem. However, the correlation coefficients were below .10, so there was truly no significant association.

The items swim and throw did not correlate with any task from the ZNA. The report on swimming might be influenced more by the environment, such as the opportunity to learn swimming than the actual motor competence. The ZNA did not include object control, so it was not expected that throwing would correlate high with other FMS. The analysis separated for different age groups revealed some weak and moderate, significant correlation but confirmed altogether the weak association between FMSQ and ZNA.

The internal consistency of the FMSQ was rather low indicating that single items may not measure a unique construct. Given the diversity of the items asked, this finding was expected. As we also examined and reported results of single items, low internal consistency is no strong limitation for the study. An explanation for the generally weak correlations could be that the variability within the items was sometimes too small, for example, only 0.5% reported that their child could not climb stairs, but over 90% could climb the stairs without holding the banister. It could also be that parents do not provide valid data on children’s FMS performance level during the preschool years. Other studies have shown that parental reports on motor milestones in the first 2 years are a valid marker of motor development of infants [4, 15] indicating that parents deliver valid data about the motor competence of their child. This current study shows that this may not be the case as children grow older. The parental report may also not be valid because parents may not have had the opportunity to observe the questioned FMS if they do not spend much time with their children or spend time doing activities for which no FMS are needed. However, only for the item balancing parents reported not to know if their child can balance. Further, certain items such as ride or swim can be related to not having much opportunity to swim or ride rather than be an indicator of the motor skill level. The low correlations between the questionnaire items and the ZNA outcomes may be explained by our sample that included only typically developing children. There is evidence that parental reports in clinical populations are more valid [20]. Miller et al. [20] reported in a sample of 2 years old with developmental disorders (e.g., autism, global developmental delay, developmental language disorder) that parental report on language and fine motor skills did not differ significantly from the measured skills. Finally, the asked and tested motor skills were possibly too different in their nature. Although all skills are indicators for gross motor competence, the asked items are more complex motor skills, while the tested skills are more basic motor skills. In this context, it has to be mentioned that the ZNA3–5 primarily measures motor abilities, which—to a large extend—cannot be practiced and are not dependent on the environment [10]. In fact, a motor test focusing more on skills may correlate higher with the parental report presented in this study.

Some limitations of the study need to be mentioned. For instance, the variability within certain items was small (e.g., for the item balance, 33% of the parents reported not to know the level of performance). The internal consistency of the FMSQ was rather low. Moreover, we did not ask if the child had the opportunity to do all the tasks. However, the percentage of children not able to swim or ride a bike was according to age.

In sum, parental report presented in this study did not provide valid data on motor development, tested by the ZNA3–5 in preschoolers. A parental report may be a valid instrument, if the items are further adapted: The items should not be strongly dependent on the environment of the child (e.g., opportunity to swim) and better differentiate between children with varying motor skills within the same age group (e.g., more categories per item). However, whether parental questions really allow a valid description of motor development and identification of children with delayed motor development remains unclear. Thus, we conclude that the evaluation of FMS performance level in healthy preschool children by their parents may not replace an objective examination of the motor skills with standardized instruments. Parental report may be considered as a screening instrument in combination with an objective examination. Given the importance of motor development due to the interrelatedness with other developmental domains and social interactions, efforts to facilitate the best possible assessment of motor development should be pursued.

## Abbreviations

FMS: Fundamental motor skills
FMSQ: Fundamental motor skills questionnaire
SPLASHY: Swiss Preschoolers’ Health Study
ZNA3–5: Zurich Neuromotor Assessment 3–5
